# Osteoporosis

**Published:** 1896-04

**Authors:** George C. Faville

**Affiliations:** Norfolk, Va.


(To be concluded in the May number.)

---

### OSTEOPOROSIS.[1]

BY GEORGE C. FAVILLE, D.V.M.,
NORFOLK, VA.

THE term osteoporosis, it seems to me, is more or less a misnomer. Indicating as it does a porous condition of bone fails to convey any accurate idea of the pathological condition found in this disease. There is upon the part of many writers a tendency to confound the disease which we commonly speak of as osteoporosis with other diseases which may result in a porous condition of the bone. For instance, the mycelium threads of actinomyces, as they affect the maxillary bones of cattle, produce a condition which might quite readily be, and probably quite often is, mistaken for osteoporosis, when the microscope would demonstrate at once the true nature of the disease.

I am well aware that I shall open a wide field for discussion by the position that I shall take; but discussion is what we want, and the aim of my paper is to form a groundwork for intelligent controversy. I think that there has been a great deal of unnecessary hairsplitting in separating this diseased condition from those other similar, and to my mind, identical diseases, osteomalacia and rhachitis. I am of the opinion that it would be much nearer accurate nomenclature to use the term rhachitis to cover all the other forms constituting the so-called diseases osteoporsis, osteomalacia, rickets, etc. The most of the literature upon this subject we find in works upon human pathology, and this is unfortunate from the fact that the human subject does not offer the field for careful pathological study that

[1] Read before the Virginia State Veterinary Medical Association, January 2, 1896.

is offered when the subjects are from some of the lower orders. A multiplicity of names tends to confuse, and it seems to me that the writings of most of our authors are confused by this very cause. I think that we are warranted in assuming that the rickets of the dog or pig and the osteoporosis of the horse are nearly enough alike in their pathology and etiology to be, *generically* at least, classed together, under the term rhachitis. Prof. W. L. Williams, in a discussion of this subject in a paper read by him before the U. S. V. M. A., held in Washington, in September, 1891, discussed this subject in a masterly manner, and I can do no better than to commend his paper to your careful consideration.

Among the writers upon human medicine it seems to be pretty well agreed that rickets is a persistence of the soft condition of the bones of the young animal, owing to a failure in the process of calcification, while osteomalacia is more a condition of age, and results in a resoftening of the bones by a process of decalcification. Among the various descriptions of osteoporosis we find one writer describing a condition which has been described by someone else under the term rickets, and another describes as the same disease those symptoms which by someone else are described as osteomalacia. We must have something else than age upon which to base a classification. The pathological conditions noted in this disease do not, it seems to me, give sufficient grounds upon which to differentiate. Billroth in his *Surgical Pathology*, in writing of predisposing causes of fractures, writes of rickets, "which is due to deficient deposit of lime-salts in the bone." Again, of rhachitis and osteomalacia, he emphasizes the point that rhachitis is a disease peculiar to childhood, and from his description of rhachitis and osteomalacia leads one to think that this differentiation is based rather upon the age of the subject than upon the differences in pathological condition. Williams, in his *Principles of Veterinary Surgery*, classes osteoporosis as a non-inflammatory disease of bone. If he means by that a disease of bone with no symptoms of inflammation present, I do not think his classification will hold. His definition of inflammation is "a perverted nutrition of a living part, the effect of irritation or injury." Taking this definition as correct and comprehensive, the classification would perhaps hold, but it is certain that we find present in this disease those symptoms found where there is inflammation. Heat, generally with redness, pain, and swelling are always met with. It would be more nearly correct to consider this disease inflammatory, the effect of a perverted nutrition.

The etiology of osteoporosis is a question of much dispute. There can be no doubt that some fault in the hygienic surroundings is the predisposing cause. Just what that fault is it is difficult to determine. We find the disease affecting young and aged animals; in horses and colts that are running on pasture with no apparent fault in the hygienic conditions, and again in stables supposed to have been constructed according to the most modern rules of sanitary science. It seems to me that there is often a constitutional diathesis, not necessarily hereditary, which predisposes to rhachitic troubles. Many times, without doubt, we must look to the unsanitary construction of stables for the predisposing cause, especially in those extensive outbreaks which appear at times in some portions of the country in an enzoötic form. But from the fact that the disease appears in horses that are not stabled or worked, that run upon pastures or the open prairie of the West, particularly some portions of the Mississippi Valley, we cannot conclude that the unsanitary construction of stables is the only predisposing cause.

I can do no better in discussing the symptomatology of this disease than to describe the course of a case which recently came under my care, a bay filly, one year old, standard bred. I knew this colt during her whole life. She was kept upon the place where she was foaled until she died. The place where she was raised is a small dairy-farm within the city limits. The drainage is exceptionally good, the farm being nearly surrounded by tide-water, and lying on a high knoll above high water. There are seven or eight horses kept on the place, and all are fed and watered from the same bins and wells. The stabling is comfortable. This filly, as is a full sister one year older, was always kept in an outside box-stall, with dry floor and windows opening directly outside. During the summer she was always out during day and night when the weather was good, on good clover pasture, with grain feed twice a day. On the 3d day of last April she was led to a blacksmith-shop near my office to have her feet trimmed. She was a little restless and would pull way from the blacksmith when he began to rasp the foot. Upon giving a sudden pull she completely dislocated the left scapulo-humeral joint. I was standing near watching the blacksmith at his work, and knew that the pull she made was a slight one, he not attempting to hold her. I at once reduced the dislocation, and with surprising ease, and in about two weeks she was as well as ever. About the last of May my attention was called to this filly again. She had not been doing well, and, while eating as well as the other stock, was not thrifty. I found a slight glairy discharge from both nostrils. The bones of the whole facial region seemed to be thickened, and there was marked dyspnœa, evidently from the thickened turbinated bones. At this time there was no lameness. In about a week the discharge from the nose had increased, and respiration had become more difficult, and I found her decidedly lame in the right foreleg. The next day she was lame in both forelimbs. In two or three days she was lame in the hind-legs, the difficulty in front having left her. The swelling of the facial bones kept increasing until respiration became fearfully painful. Her food could be but imperfectly masticated. The teeth seemed sore, although the gums showed no inflammation.

About the middle of June she died, and I made a post-mortem examination. The only lesions I could find were in the osseous system. The superior maxillary, nasal, and turbinated bones were greatly thickened. The periosteum was easily separated, and the whole bone was soft and spongy; I could readily press a small scalpel into the substance of the bone at any point. I made an attempt to clean them by boiling. I found them so soft that I could keep them in no way except in alcohol. They seemed to have completely lost their osseous character. I found the long bones at their extremities showed the same condition to a greater or less extent. This case in its symptoms and developments was typical of the disease as I have seen it. I carefully studied the condition under which this colt was kept. The food was of the best, good hay and grass, with a daily run at pasture during good weather, and dry, well-ventilated stabling at night and during bad weather. Every attention was given her in the hopes of producing a speedy mare. I can account for this case in no way except by assuming that there was some fault with the process of assimilation. Evidently all the necessary elements for bone-formation were present in the food. Other colts of the same age, kept under the same conditions and fed in the same way, are thrifty and developing nicely. The digestive functions seem to be perfectly performed. There certainly, in this case, was not, in fact I have never known of a case where there was, anything to indicate a hereditary diathesis. Undoubtedly the materials necessary for complete bone-formation were present and in proper proportion for assimilation, but either are not assimilated or by some occult action after assimilation are reabsorbed.

In order to get the various views of our members, I prepared a series of questions regarding this diseases which I sent to each member. Several replied that they had never seen cases of this disease in their practise in this State. The questions asked were as follows:

1st. To what extent have you observed osteoporosis in this State?

2d. What proportion of cases have been aged and what young animals?

3d. Were the animals stabled? If so, please give details as to ventilation, drainage, dampness, character of food and water. If on pasture, please state character of pasture, whether dry or marshy, and kind of grass and water.

4th. At what age have you generally seen the disease, and in your opinion what is the cause of the disease in the cases you have seen?

5th. What line of treatment do you find best?

6th. What percentage of recoveries?

7th. The uncertainty of our knowledge regarding this disease makes it more than interesting. Kindly send such information as you may have upon the peculiarities of its appearance in your locality?

